# Cryptic Diversity of African Tigerfish (Genus *Hydrocynus*) Reveals Palaeogeographic Signatures of Linked Neogene Geotectonic Events

**DOI:** 10.1371/journal.pone.0028775

**Published:** 2011-12-14

**Authors:** Sarah A. M. Goodier, Fenton P. D. Cotterill, Colleen O'Ryan, Paul H. Skelton, Maarten J. de Wit

**Affiliations:** 1 Department of Molecular and Cell Biology, University of Cape Town, Rondebosch, Cape Town, South Africa; 2 Africa Earth Observatory Network (AEON), University of Cape Town, Rondebosch, Cape Town, South Africa; 3 South African Institute of Aquatic Biodiversity (SAIAB), Grahamstown, South Africa; 4 Africa Earth Observatory Network (AEON), Earth Stewardship Science, Nelson Mandela Metropolitan University, Port Elizabeth, South Africa; University of Otago, New Zealand

## Abstract

The geobiotic history of landscapes can exhibit controls by tectonics over biotic evolution. This causal relationship positions ecologically specialized species as biotic indicators to decipher details of landscape evolution. Phylogeographic statistics that reconstruct spatio-temporal details of evolutionary histories of aquatic species, including fishes, can reveal key events of drainage evolution, notably where geochronological resolution is insufficient. Where geochronological resolution is insufficient, phylogeographic statistics that reconstruct spatio-temporal details of evolutionary histories of aquatic species, notably fishes, can reveal key events of drainage evolution. This study evaluates paleo-environmental causes of mitochondrial DNA (mtDNA) based phylogeographic records of tigerfishes, genus *Hydrocynus*, in order to reconstruct their evolutionary history in relation to landscape evolution across Africa. Strong geographical structuring in a cytochrome b (*cyt-b*) gene phylogeny confirms the established morphological diversity of *Hydrocynus* and reveals the existence of five previously unknown lineages, with *Hydrocynus tanzaniae* sister to a clade comprising three previously unknown lineages (Groups B, C and D) and *H. vittatus*. The dated phylogeny constrains the principal cladogenic events that have structured *Hydrocynus* diversity from the late Miocene to the Plio-Pleistocene (ca. 0–16 Ma). Phylogeographic tests reveal that the diversity and distribution of *Hydrocynus* reflects a complex history of vicariance and dispersals, whereby range expansions in particular species testify to changes to drainage basins. Principal divergence events in *Hydrocynus* have interfaced closely with evolving drainage systems across tropical Africa. Tigerfish evolution is attributed to dominant control by pulses of geotectonism across the African plate. Phylogenetic relationships and divergence estimates among the ten mtDNA lineages illustrates where and when local tectonic events modified Africa's Neogene drainage. Haplotypes shared amongst extant *Hydrocynus* populations across northern Africa testify to recent dispersals that were facilitated by late Neogene connections across the Nilo-Sahelian drainage. These events in tigerfish evolution concur broadly with available geological evidence and reveal prominent control by the African Rift System, evident in the formative events archived in phylogeographic records of tigerfish.

## Introduction

As a dominant determinant of continental topography and drainage, tectonism demonstrates profound controls over the biogeography of landscapes [1]–[5]. The biogeography of extant freshwater fishes represents responses to landscape evolution because their ecology encompasses responses to habitat changes. Evolutionary patterns of African fish faunas illustrate these tight causal linkages, where species biogeography corresponds closely with events that have reconfigured drainage networks throughout the Cenozoic [6]–[11]. The corollary follows that patterns of genetic variation represented in distributions of extant populations constitute spatial-temporal signatures of landscape history. Thus, it is expected that biogeographical patterns in these aquatic species constitute a historical archive which can resolve key events in the geomorphic history of drainage basins [12]. As recently demonstrated for Quaternary landforms in New Zealand, the interlinked histories of fishes and drainage systems can be reconstructed to quantify the tempo and mode of shared events in biogeographical and geological history [13], [14]. Beyond resolving biodiversity dynamics, increasingly powerful methods in phylogeography can quantify the spatio-temporal details of shared events in evolutionary histories of biota and landscapes [12], [14], [15], [16].

**Figure 1:**
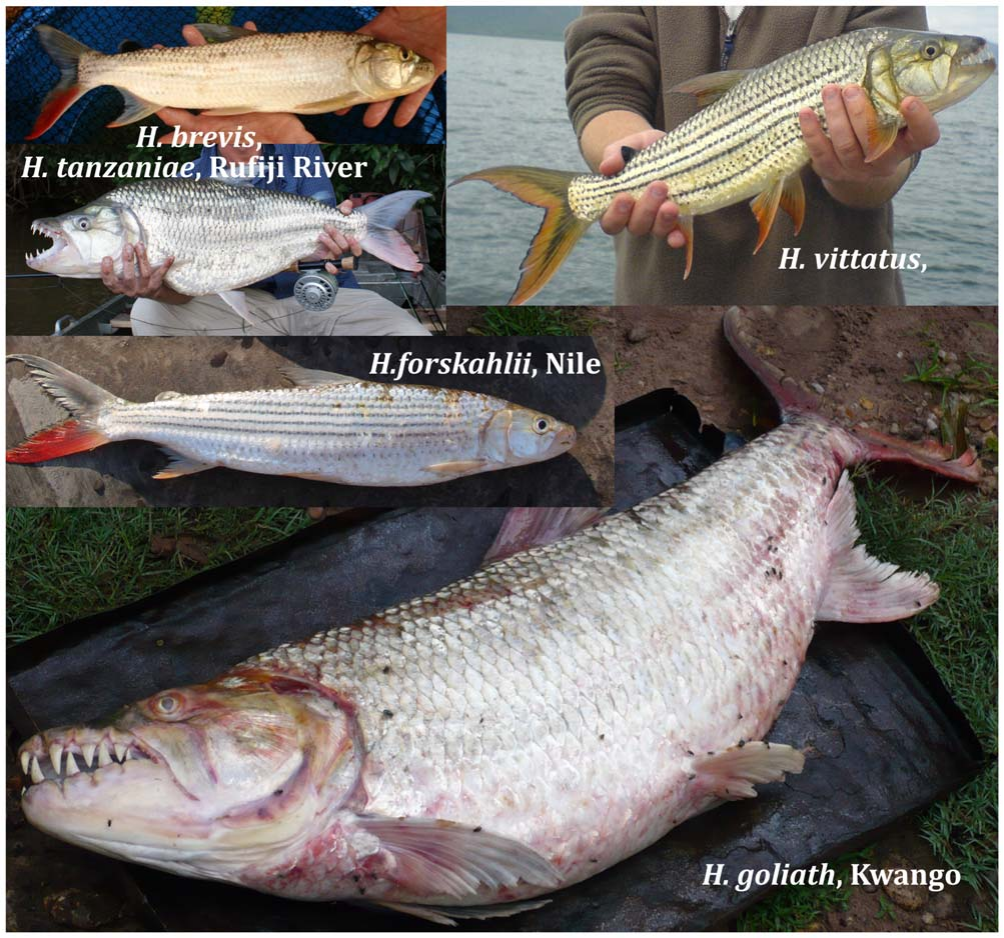
The five recognised and named species of *Hydrocynus*. From bottom (clockwise): a) *H. brevis,* b) *H. vittatus,* c) *H. goliath,* d) *H. forskahlii* and e) *H. tanzaniae*. Photo credits: a) Stan Nabozny, b) Ryan Clark, c) Mike de Wit, d) Dirk Neumann, e) Keith Clover.

**Figure 2:**
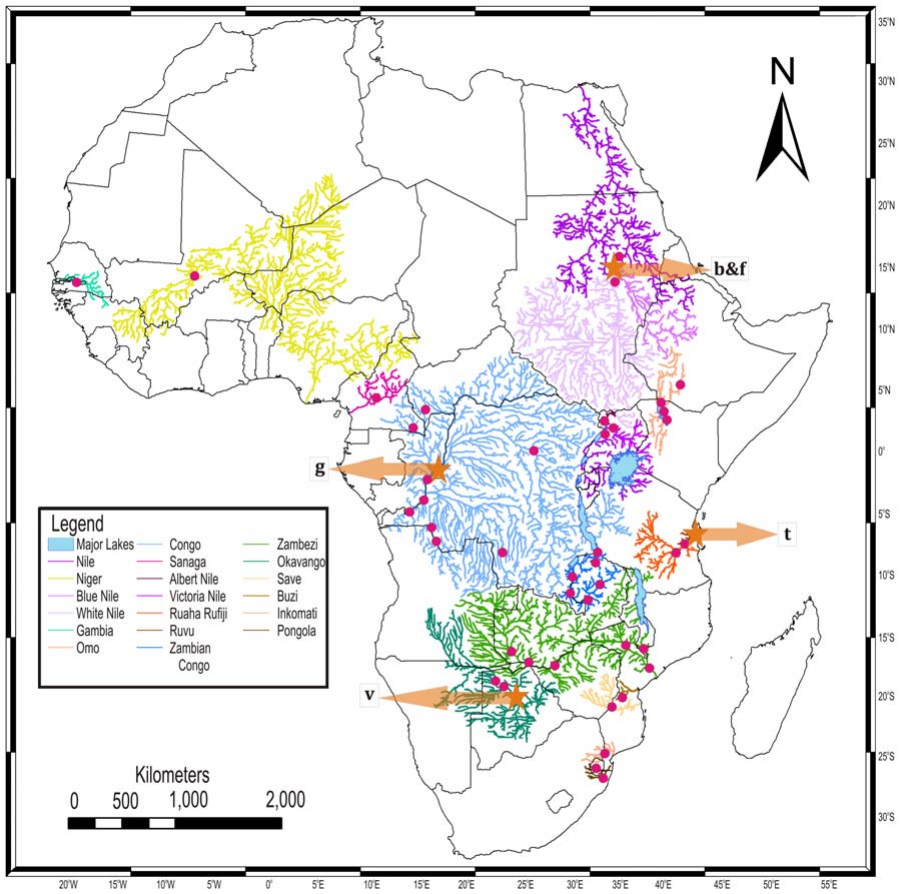
Sample localities of *Hydrocynus* in relation to the principle drainage basins of Africa. This map was constructed with ArcMap 9.3 from the HydroSHEDS digital river database [103] and the AEON Africa rivers database [65], [104]. Country boundaries and labels are included for geographical context. Arrows designate type localities [in square parentheses] for the five recognized species of *Hydrocynus*: b = *H. brevis* (Günther 1864) [White Nile]; f = *H. forskahlii* (Cuvier 1819) [White Nile]; g = *H. goliath* Boulenger 1898 [Main Congo, Mbandaka]; t = *H. tanzaniae* Brewster 1986 [Lower Ruvu River]; v = *H. vittatus* (Castlenau 1861) [Okavango Delta]. The northern, southern and eastern boundaries of the Congo basin coincide with the tectonically active Cameroon Rift and Central African Thrust Zone [31], [32], Southern Equatorial Divide [33] and East African Rift System (EARS) [17], respectively.

**Figure 3:**
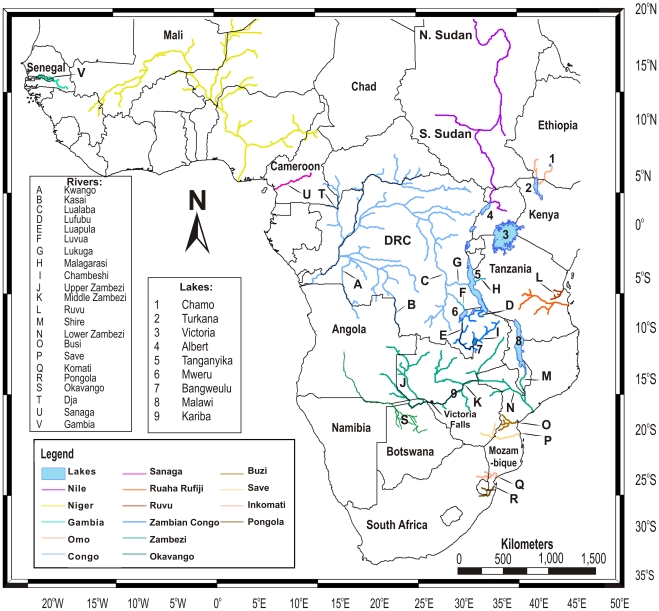
Map of the relevant lakes and rivers that frame this study. This map depicts the geographical relationships of the main rivers and lakes relevant to the biogeography and evolution of *Hydrocynus*, and localities mentioned in the text. This map was constructed using ArcMap 9.3 from the HydroSHEDS digital river database [103] and the AEON Africa rivers database [65], [104]. Main rivers and lakes depicted in the legend are labeled with letters (rivers) and numbers (lakes) respectively. Country boundaries and labels are included for geographical context.

This study evaluates how selected fish species perform as biotic indicators of landscape evolution, by testing their application to resolve details of geomorphological evolution and paleogeography. The reconstruction of phylogeographic events in the evolutionary history of tigerfishes, genus *Hydrocynus* (Cuvier 1816) (Figure 1), enables a test of how biotic history relates to that of evolving African landscapes, and especially episodes of tectonism in Rift systems. Here we evaluate how rearrangements of Africa's drainage through the late Cenozoic - reviewed by Goudie [17] - interfaced with the evolution of tigerfishes. The geographic coverage encompasses tropical sub-Saharan Africa. Representing a total of 23 principal rivers within 15 geographically isolated drainage basins, this study represents nearly all populations of *Hydrocynus* (Figures 2 and 3) within the timeframe of the Neogene period (0–23 Ma). The tigerfishes selected in this study as biotic indicators of drainage evolution are freshwater teleosts of the Order Characiformes [18], [19]. These keystone, open-water predators prey on fishes up to approximately 40% of their body length [9], [20], [21] (Figure 1). The earliest *Hydrocynus* fossils, dated as Late Miocene, are from the Chad, Maronga, Turkana and Semliki basins of north and east Africa [22], [23], [24], where tigerfishes still occur in extant wetlands [11]. Although *Hydrocynus* exhibits a Pan-African distribution, their absences from particular drainage systems highlight curious range gaps, recognized decades ago, which have yet to be adequately explained [10], [25], [26]. Tigerfishes are largely absent from east Africa, east of the Albertine Rift, pertinently from Lake Victoria. Their absence from Lake Malawi is equally anomalous. Yet, their occurrence in the Omo and Albert Nile drainage systems stands out, as does *H. tanzaniae* isolated in the Ruvu and Rufiji-Ruaha drainage basins (eastern Tanzania) from those in the Congo, Nile and Zambezi basins [10], [11]. Pertinently, late Miocene fossils from Paleo-Lake Manonga (northern Tanzania) reveal an early presence on the Tanzanian plateau [22]. . Bell-Cross [6] proposed that relative timings of knickpoint evolution, particularly rapids and waterfalls (exemplified in the Victoria Falls), had contained fish dispersals in south-central Africa (Figures 2 and 3). This mechanism, driven by landscape evolution, potentially explains a contingent history of range evolution in tigerfishes.

Additional to their patchy distribution across Africa's drainage basins, the ecology of *Hydrocynus* is of key relevance to this phylogeographic investigation. Tigerfishes are restricted to relatively warm, fast-flowing and/or open, oxygen-rich habitats in large rivers and lakes [10], [11] as recently confirmed by stable isotope signatures in fossil and extant *Hydrocynus* [27]. Because their stenotopy confines them to larger river channels and open lakes [27], [28], tigerfishes are absent from headwater marshes and streams along watersheds. This pattern of habitat selection is exemplified in the Upper Zambezi River and Okavango Delta where tigerfish favour deep, flowing channels, avoiding more anoxic and shallow backwaters and lagoons [20], [28], [29]. The ecological dependence of *Hydrocynus* on well-oxygenated freshwaters contrasts against other fishes (e.g. *Clarias gariepinus*) which, being more eurytopic, can tolerate a far wider range of ecophysiological conditions [28], [30]. This enables such eurytopes not only to cross shallow watersheds but also to persist in marginal aquatic habitats even where main rivers dry out. Although the premise has yet to be rigorously tested, these differences between eurytopes and stenotopes in aquatic habitats, are predicted to be reflected in significant differences in phylogeographic structuring of respective species within and across drainage basins [12]. Therefore it is hypothesized that tigerfish evolution has tracked formative changes in the topology of major river channels, and equally reflects an inherent sensitivity to changes in habitat quality associated with episodic climate change. Moreover, it follows that tigerfishes require links in the form of major river channels (as opposed to shallow ephemeral connections between headwaters across watersheds) to disperse between neighbouring drainage systems.

If the tectonism that reconfigured African drainage basins has had only marginal impacts on ancestral populations of tigerfishes, then rifting history will be decoupled from the tempo and mode of *Hydrocynus* evolution. Conversely, contingent on spatio-temporal resolution, we can expect phylogeographic records of *Hydrocynus* to preserve fingerprints of paleogeographic history, which would testify to impacts of tectonism across the African plate. If subsequent dispersals are prevented, new drainage divides forged by tectonism can be expected to become foci of biotic disjunction. We should equally expect distinct phylogeographic structuring across dispersal barriers within larger drainage basins, which reflect reduced admixture between neighbouring populations.

In this respect, the boundaries of the Congo basin are of focal interest, because these watersheds were forged by diastrophism and/or epeirogeny. The northern, eastern and southern boundaries of this basin are the Cameroon Rift and Central African Thrust Zone [31], [32], the East African Rift System (EARS) and Southern Equatorial Divide [33]. Moreover, geochronological evidence for the Central African Thrust Zone and Albertine Rift (western arm of the EARS) reliably constrains when volcanism and tectonism formed these hydrological boundaries around the Congo basin. So it delimits when first and second order rivers of this basin were redirected and/or impounded by faulting and uplift events (Figure 2). Therefore we can employ phylogeographic statistics to test whether or not evolutionary events in *Hydrocynus* were coupled with this drainage disruption, linked with propagation of rifting across the African plate (exemplified by the formation of Lake Tanganyika) [12], [34], [35], [36].

Here we use analyses of genetic variation and molecular dating methods to test for geomorphic control by paleo-drainage dynamics of the evolution of *Hydrocynus*. A chrono-biogeographical approach [37], [38] structures this paper, employing phylogeographic analyses in a cross-disciplinary, geobiological framework [12]. Quantified patterns of spatio-temporal genetic variation enable tests for relative roles of vicariance and/or dispersal in shaping tigerfish diversity. A biochronological reconstruction of diversification in *Hydrocynus* frames analyses of the demographic structuring of genetic variation in a phylogeny informed by mtDNA sequence data. Molecular dating of principal nodes in the phylogeny provides critical constraints to test the relative fidelity of biotic events against tectonic events. Besides orthodox statistical tests of nodal support (bootstrapping and Bayesian posterior probabilities) in phylogenetic reconstruction, we employ statistical tests of genetic admixture and genetic variation among tigerfish lineages to evaluate influences of drainage rearrangements, and thus tectonism.

## Results

### Data and Haplotypes Summary

Our database comprises a total of 90 individuals sampled from rivers in discrete drainage basins across Africa (Figures 2 and 3). The 823 bp *cyt* b fragment obtained from 90 individuals yielded 42 haplotypes, of which 88 individuals were generated in this study (Table S1). The General Time Reversible (GTR) model [39] plus Gamma (G) was chosen for the *cyt* b data (Table 1). The *cyt* b region was found to be C and A-T rich but G poor, corresponding to the general pattern found in a study on *cyt* b evolution involving 81 genera of fish [40], and a higher proportion of transitions were also found compared to transversions.

**Table 1:** Parameters for the best-fit model (GTR+G) for *cyt b* sequence data (G = Gamma).

	*Cyt b*
**Model**	GTR+G
**Base frequencies:**	
A Frequency	0.2529
C Frequency	0.3232
G Frequency	0.1481
T Frequency	0.2757
**Rate Matrix:**	
R(a) [A-C]	5.1501
R(b) [A-G]	62.5377
R(c) [A-T]	1.6271
R(d) [C-G]	2.6663
R(e) [C-T]	21.1383
R(f) [G-T]	1.0000
**A**	0.1691

### Dated Phylogeny

**Figure 4:**
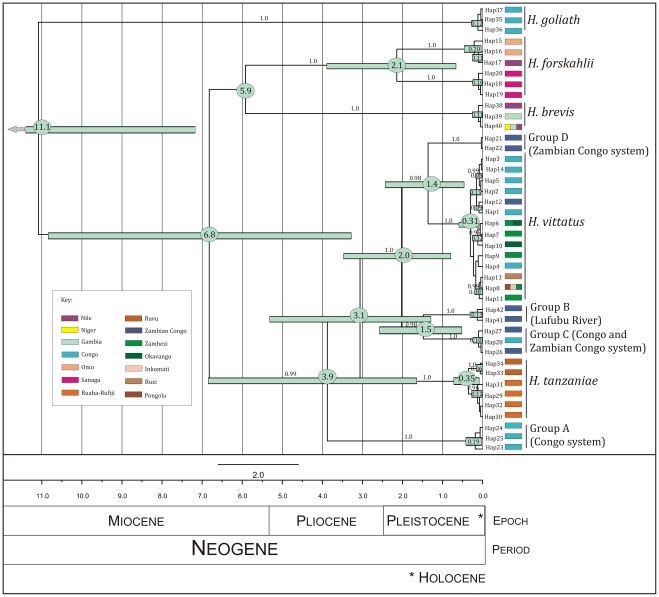
Dated Bayesian tree of the *cyt b* sequence data of *Hydrocynus* produced in BEAST, using the GTR parameters specified by Modeltest. Only posterior probabilities over 0.90 are shown. Error bars indicate the 95% confidence intervals on the node dates; numbers inside the bars are the dates estimated by BEAST; those inside ovals are discussed in the text. The horizontal axis depicts time before present in Millions of Years (Ma). The colour coding of *Hydrocynus* lineages corresponds to the drainage system(s) represented in each haplotype.

BEAST [41], Mr Bayes [42] and PAUP [43] all produced trees with consistent major branching orders that exhibit a strong topological congruence (Figures S1, S2 and S3, respectively). The dated phylogeny (Figure 4) exhibits equally close congruence, and illustrates divergence dates in millions of years (Ma) with confidence intervals (CI) (Tables 2 and 3). A total of ten unique evolutionary mtDNA lineages were recovered with high support (bootstrap and posterior probability). Beyond recovering lineages that correspond to the five species recognised by morphological characters [44], [45] these data reveal the existence of previously unrecognized diversity within *Hydrocynus*, with the ranges of three of these lineages confined within the extant Congo drainage basin (Table 4, Figures 2, 3 and 4).Until such time that this taxonomic complexity of *Hydrocynus* is formally characterized, these previously unrecognized lineages are distinguished as follows (Table 4):

- Group A is represented by Congo basin samples from the Kwango, the main Congo, upstream of Kisangani and the Lulua River (a tributary of the Kasai River);
- Group B consists of two samples from the Lufubu River (a tributary of Lake Tanganyika) collected alongside a series of *H. vittatus*;
- Group C comprises a population that is most abundant within Lake Mweru, with additional samples from Lake Bangweulu and the Dja River (a tributary of the Sanaga);
- Group D is composed of samples from the Luapula River, Lake Mweru, Lake Bangweulu and the Chambeshi River. This lineage is sister to *H. vittatus*;
- Group E is comprised of samples from the Sanaga River, southwest Cameroon, and is sister to *H. forskahlii sensu stricto*.

**Table 2:**
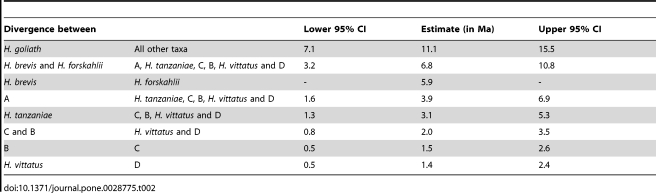
Estimated timing of the divergence events between main lineages recovered in the dated BEAST tree including the 95% CI's.

**Table 3:** Estimated timing of the divergence events within lineages recovered in the dated BEAST tree including the 95% CI's.

Main divergence within	Lower 95% CI	Estimate (in Ma)	Upper 95% CI
*H. forskahlii*	0.6	2.1	3.9
*H. tanzaniae*	0.1	0.35	0.7
*H. vittatus*	0.05	0.31	0.6

**Table 4:** Overview of all ten mtDNA lineages recovered in the phylogenetic tree (*cyt b* data) in relation to their geographical distributions.

Lineage	Species Complex	Drainage Systems of Occurrence
***H. vittatus***	Vittatus complex	Congo, Lake Tanganyika, Zambezi, Okavango, Coastal systems south of Lower Zambezi
***H. goliath***	-	Congo
***H. forskahlii***	-	Sanaga, Nile, Omo
***H. brevis***	-	Nile, Gambia
***H. tanzaniae***	-	Ruvu, Rufiji
**A**	-	Congo
**B**	Vittatus complex	Lake Tanganyika (Lufubu)
**C**	Vittatus complex	Congo, Zambian Congo
**D**	Vittatus complex	Zambian Congo
**E**	Forskahlii complex	Sanaga River, West Cameroon

Drainage systems and main rivers are mapped in Figures 2 and 3, respectively.

Hereafter, groups B, C, D and *H. vittatus sensu stricto* (s.s.) are designated the Vittatus complex.

The discovery of cryptic Group E, confined within the Sanaga River, exemplifies the importance of representative geographical sampling to survey all principal drainage basins. This relatively deep divergence at 2.1 (CI: 3.9–0.6) Ma between these two allopatric lineages raises interesting issues for the prevailing taxonomy [44], as it reveals that *H. forskahlii* comprises a species complex. This monophyletic Sanaga clade is isolated from all other representatives of *H. forskahlii* in the Nile River and Lake Chamo, Omo system (southern Ethiopia). Its evolution can be attributed to divergence within the *H. forskahlii* lineage from the parent population across an ichthyofaunal province, possibly in response to drainage evolution. *H. forskahlii* and *H. brevis* showed a poorly supported sister relationship in this dated phylogeny, which was not recovered in the MrBayes tree (Figure S1). As the Birth-Death Speciation prior was used to construct this dated BEAST tree, the difference in tree topology could be a function of this algorithm. Due to the weak support for this topology, no confidence limits on the dating of this event are given, making its estimate of the divergence timing between these two lineages questionable. So this event will not be included in the discussion of landscape evolution.

A very recent (Middle Pleistocene) divergence in the *H. tanzaniae* lineage (0.35 CI: 0.7–0.1 Ma) was also discovered, with strong nodal support, which distinguishes samples from the Ruvu River from individuals sampled in the Kilombero River (part of the Rufiji-Ruaha system) (Tables 2 and 3).

Within *H. vittatus*, three well supported groups were recovered in the Bayesian tree. These groups consisted of samples from a) the Congo and Lufubu Rivers, b) the Upper Zambezi and Okavango Delta, and c) the Middle-Lower Zambezi and Shire Rivers and the coastal populations. These groups reveal strong phylogeographic structuring across drainage systems, and their detailed explanation will be explored in greater detail in a separate paper [Goodier *et al.* unpublished data]. Several haplotypes shared between different drainage systems and different rivers within the same system, as seen above, are explored further in the Discussion.

### Descriptive Statistics

Nucleotide and haplotype diversities [46] for the *cyt b* data set (Table S2) show high haplotype diversities (Hd) and moderate to high nucleotide diversities of *H. vittatus*, *H. forskahlii*, *H. tanzaniae* and Group A, indicating either stable populations with large, long term effective population sizes or admixture of historically isolated populations. Group B also shows the abovementioned pattern but, since it consists only of two individuals, this could be a sampling artefact and insufficient samples preclude meaningful data analysis of this lineage. *H. goliath*, *H. brevis* and Group C have a moderate to high Hd but a low nucleotide diversity, which indicates rapid population growth from historically small effective population(s). Very low nucleotide and haplotype diversities in Group D indicate that this lineage has experienced a severe bottleneck in its recent evolutionary history.

### Population Structure and Demographic History

The genealogical species index (GSI) values [47] were all 1 (p<0.005), which confirms that all the lineages comprising the phylogeny are monophyletic and on independent evolutionary trajectories. GSI results of *H. vittatus*, comparing encompassing drainage basins (Congo v.s. Zambezi), testify to significant structuring (0.61, p = 0.017 v.s. 0.52, p = 9.999e-05, respectively), and correspond to a suite of AMOVAs that reveal significant geographic structuring in genetic variation. The ‘species level’ AMOVA [48] of grouped lineages showed that 97.4% of the variation was among populations and only 2.6% was within populations, all with high population specific ΦST values [49] (Table S3), which points to substantial genetic differentiation of evolutionarily distinct lineages. For *H. vittatus*, when the rivers or lakes of collection were considered independently (oneway AMOVA), just over 46% of the total sequence variance was explained by differences among collection sites while approximately 53% was accounted for by variation among individuals within sites. The hierarchical AMOVA reveals geographical boundaries structured variation across collection sites (Table 5). A pertinent example is the Victoria Falls, here confirmed as an important barrier to tigerfish dispersal in the Zambezi basin [6], [8], which explained nearly 44% of the variance, a similar amount explained by collection sites alone. Grouping the samples according to river system and broad drainage system explained similar amounts of variation among groups (25.71% and 26.68%, respectively), both less than half that explained by grouping by boundaries. In summary, this structuring of genetic variation points to historical controls by barriers to dispersal.

**Table 5:** Geographically structured analysis of molecular variance (AMOVA) using *cyt b* sequences for 26 *H. vittatus* individuals.

	% of variation (df)		
	Among groups	Among populations within groups	Within populations
All samples	46.52 (5)	n/a	53.48 (20)
Groups			
A – Broad drainage systems (Congo vs. Zambezi)	25.71 (1)	27.49 (4)	46.80 (20)
B – River systems (Congo and Kwango vs. Zambezi and Okavango vs. Eastern Coastal drainage)	26.68 (2)	23.46 (3)	49.86 (20)
C – River groups (Congo and Kwango vs. Upper Zambezi and Okavango vs. Middle and Lower Zambezi, Lower Shire vs. Eastern Coastal drainage)	43.74 (3)	5.71 (2)	50.55 (20)

Variation is reported as a percentage of the total with degrees of freedom (d.f.).

Tajima's D [50] and Fu's Fs [51] tests of selective neutrality examine the frequencies of mutations in order to detect deviations from the neutral model [52]. Due to the small sample sizes (2–10 individuals) for all the lineages, excluding *H. vittatus*, it is not surprising that neither of these tests of selective neutrality were significant since the ability to detect deviations from the neutral model increases with sample size (Table S4]. Only *H. vittatus* had a significantly negative value (−6.2153; p<0.005) for Fu's Fs, which is more sensitive to recent population expansions [53]. This result indicates an excess of low-frequency mutations, caused by demographic expansion or selection. However, as *cyt b* is a protein coding gene, it could be under selection to retain its functionality, resulting in the significantly negative Fu's Fs.

**Figure 5:**
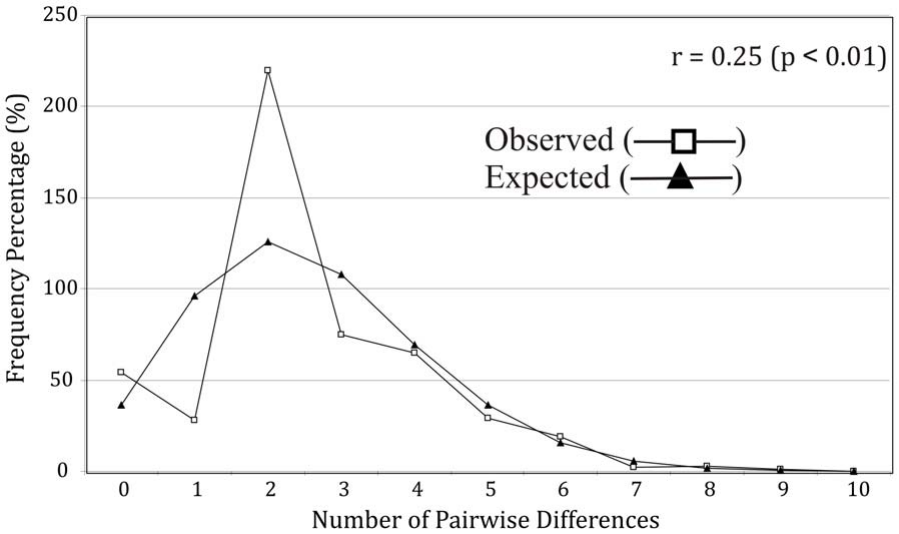
Mismatch distribution and ‘raggedness’ index (*r*) for the *cyt b* data of *H. vittatus*. Observed and expected pairwise differences based on 1000 replicates.

Since *H. vittatus* alone had a significant value for Fu's Fs, only its mismatch distribution was calculated (Figure 5). The ‘raggedness’ index was high (r = 0.25) and significant (p<0.01), indicating a poor fit of the observed and expected mismatch distributions [54]. The observed multimodal distribution, which is characteristic of a historically stable population, is supported by the high nucleotide and haplotype diversity in *H. vittatus*, which indicates a stable population with a large, persistent, effective population size. However, this can also indicate admixture of historically isolated populations but the low level of genetic distance within *H. vittatus* does not support a historically stable population.

## Discussion

This study provides the first complete molecular phylogeny of *Hydrocynus*, which incorporates all described species, with representative geographical coverage. Moreover, spatio-temporal resolution of diversification in *Hydrocynus*, revealed by molecular dating of cladogenic events and structuring of genetic variation, points to pervasive control of landscape evolution, within and across extant drainage basins. These implications, pertinently as tests of tectonic control, are discussed in detail below. However, first we set in place the platform of taxonomic and phylogeographical knowledge informed by this phylogeny and associated phylogeographic statistics.

### Taxonomic Implications

Besides representative geographical coverage, the *cyt b* genotypes obtained in this study (Table S1) include topotypical representatives of all five recognized *Hydrocynus* species (Figure 2): from the Nile river (*H. forskahlii*), Khartoum, Nile (*H. brevis*), Main Congo river (*H. goliath*), Ruvu and Rufiji rivers (*H. tanzaniae*), and Okavango Delta (*H. vittatus*) [55], [56] (Figure 2). Recovery of congruent evolutionary relationships with multiple phylogenetic methods lends confidence in the phylogeny obtained for *Hydrocynus*, with strong nodal support (bootstrap, GSI and posterior probability). The sampling representation in this study not only underscores the taxonomic representativeness of our genetic results but lays to rest any residual controversy pertaining to Brewster's [56] treatment of *H. vittatus* as a synonym of *H. forskahlii*. Beyond endorsing the previous refutation of this synonymy on morphological grounds [55], genetic evidence reveals that the marked phylogenetic divergence between *H. forskahlii* and the Vittatus complex (including *H. vittatus*) represents a relatively deep (Miocene) cladogenic event in the evolution of *Hydrocynus* (Table 2, Figures 2 and 3). Clearly, the mtDNA phylogeny produced in this study significantly improves our knowledge of the diversity of the genus and evolutionary relationships among its species. Moreover, the spatio-temporal complexity in *Hydrocynus* relationships, revealed in molecular dating of divergence events, focuses questions awaiting biogeographical explanation.

Beyond corroboration of morphological evidence for five species recognised in the current taxonomy of *Hydrocynus* [44], [45], the discovery of five previously unknown lineages (Groups A–E), each with independent evolutionary histories initiated in the Plio-Pleistocene, indicates that significant tigerfish diversity has been overlooked in Africa. We argue that these cryptic species await formal taxonomic characterization. In particular, these discoveries highlight the existence of three distinct lineages in the Congo basin, sympatric with *H. goliath* and *H. vittatus*. The discoveries of cryptic diversity in *Hydrocynus* raise poignant questions about the future management of fisheries as well as the existence of other undiscovered biodiversity in African drainage systems.

With the aim of resolving the cryptic diversity in *Hydrocynus*, current research is examining representative museum specimens of all these taxa in a multi-faceted generic revision. This revision will include formal taxonomic evaluation of all cryptic lineages revealed by mtDNA genotyping.

### Phylogeography

Viewed at a continental scale, the phylogeography of *Hydrocynus* reveals that the diversity and distribution of extant species reflects a complex history in which incidents of vicariance caused principal cladogenic events, but dispersal events expanded distributions of particular species to result in sympatry. The interplay of these processes is exemplified in the Congo basin, where vicariants (pertinently Group C) appear to have seeded the Congo basin from the south, after their evolution in geographically isolated drainage basins, formerly centred on Plio-Pleistocene lakes in the Bangweulu, Mweru and Upper Zambezi basins [12]. As advocated by the chrono-biogeographic strategy [37], [38], our analyses reveal two modes of speciation in *Hydrocynus*. Allopatry can be caused by either dichopatric speciation, where a widely distributed ancestral species became isolated across a new geographical barrier, or peripatric speciation, where founders disperse across an across an existing barrier with subsequent divergence. This dichotomy in mode of diversification reflects a critical difference in how biodiversity dynamics have interfaced with paleo-environmental dynamics [2], [3]. Moreover, relatively recent range expansions of dichopatric lineages explain the sympatry of *Hydrocynus* in the Congo basin, into which Group C and *H. vittatus* have dispersed from the Luapula (Lake Mweru) and Upper Zambezi, respectively.

So each evolutionary event in tigerfishes can be attributed to a spatio-temporally discrete episode of drainage evolution, which either isolated ancestral populations in new habitat or opened up dispersal opportunities for a particular species to expand its range. One such extreme dispersal event is testified to by recovery of a total of six individuals of *H. brevis* sharing a haplotype common to the Gambia, Niger and Nile rivers, vouched for by samples from Sudan, Mali and The Gambia (Table S1). Beyond testifying to the propensity for long distance dispersal in tigerfishes, it reveals a very recent connection of this species across the Sahelo-Sudanian ichthyofaunal region. This is consistent with the hypothesis of Roberts [57] that the Gambia and Senegal Rivers nearly dried out during the last dry climatic period (ca 0.12–0.27 Ma), and the extant Gambian and Senegalian fish faunas were re-colonised from the Niger River in the following wet climatic period (ca 0.08–0.12 Ma) [11]. This panmixis in *H. brevis* points to a relatively recent link between the Niger and the Nile [58], [59], although it is considered controversial by some geomorphologists [17]. Western African drainage systems comprise a high proportion of shared species with the Nile system [57], [58], which corroborates the recent faunal exchanges represented here in *H. brevis*. Broader sampling of these ichthyofaunal regions and further analysis is required to provide more detailed resolution on the relationships between *Hydrocynus* species across northern Africa. Important gaps in sampling coverage across west Africa includes the Volta basin, and also the Rovuma basin in east Africa.

As judged by scattered sampling locations, several thousand kilometres apart, several populations of *Hydrocynus* share haplotypes across vast distances in the Congo basin (Table S1, Figure 2). This indicates that these populations (representing *H. goliath* and *H. vittatus*, respectively) were connected and/or underwent major dispersals in the recent geological past. This is somewhat surprising due to the large size of the basin and physical barriers proposed to restrict fish dispersal (e.g. Kisangani Falls and Portes d'Enfer on the Lualaba River) [17], [60]. Nevertheless, it is interesting how knickpoints in the Luvua river [Flügel et al. unpublished data] can be invoked to have prevented upstream dispersal of *H. goliath* and *H. vittatus* into Lake Mweru, yet Group C appears to have dispersed downstream through the Luvua (as represented by its occurrence across the Congo basin, Figures 2 and 3). At least in the case of *Hydrocynus* Group D, the demonstration of shared haplotypes across the Zambian Congo drainage system corroborates Bell-Cross's [6] hypothesis that waterfalls on the Luapula River (linking the formerly isolated Lakes Bangweulu and Mweru) no longer restrict fish dispersal entirely (Figure 3).

In this respect, the high Hd of *H. goliath*, *H. brevis* and Group C points to a recent expansion in the sizes and ranges of these three populations. Correspondingly, the high haplotype diversities and high nucleotide diversities of *H. vittatus*, *H. forskahlii*, *H. tanzaniae* and Group A possibly represent recent admixture of historically isolated populations, in respective species, since sampling of each lineage encompassed a large geographical area and represents discrete rivers. The large internal genetic distance within *H. forskahlii* represents marked genetic structuring within this species complex, most notable in the deep phylogenetic divergence between Group E and topotypical *H. forskahlii*.

A large proportion of individuals from the Okavango and Upper Zambezi Rivers share a haplotype confirming an intermittent connection between the Okavango and Upper Zambezi systems [6], [57], [61]. A haplotype dominating *H. vittatus* individuals from coastal populations, south of the Lower Zambezi, is shared with an individual from the Middle Zambezi. The presence of the same haplotype in this large area, alongside its high abundance in the coastal rivers (Busi, Pongola and Save) is consistent with their recent colonisation from a Middle and Lower Zambezi source population.

The estimated divergence dates on the dated *cyt* b tree exhibit fairly large confidence intervals (e.g. 11.1 (CI: 15.5–7.1) Ma on the divergence between *H. goliath* and all other taxa. This most likely reflects the use of only one calibration point and a universal estimate of substitution rate in teleost *cyt b*. However, despite this large uncertainty, the estimated dates provide reliable evidence to discuss the evolutionary history of *Hydrocynus* since even approximate estimates can be used to explain biogeographical history [37], [38], [62], [63].

With the exception of Group E in the Sanaga river (Figure 3), all these newly discovered cryptic lineages occur in sympatry with at least one described species of *Hydrocynus*. It is still unclear if these sympatric lineages occupy different ecological niches and/or breeding habitats, given the evidence of their allopatric origin, within ancestral distributional barriers, before subsequent dispersals resulted in the sympatry seen today. This discovery endorses the need for morphological, ecological and behavioural studies of *Hydrocynus* to evaluate the hypotheses as to whether different niches (habitat selection) or breeding biology explain the reproductive isolation represented in these lineages of tigerfishes.

### Biogeography and Geotectonics

Our phylogeographic results reveal key spatio-temporal details of how divergence events and demographic responses of tigerfishes have played out across African drainage systems over the late Cenozoic. These cladogenic and dispersal events testify to the marked sensitivity of *Hydrocynus* to the geomorphic evolution that fragmented and reconfigured drainage basins. This biogeographical resolution corroborates that obtained for galaxiid fishes and drainage evolution in the Southern Alps, New Zealand [13], [14]. Collectively, they demonstrate how combined geological and genetic data provide the keys to unravel the histories of drainage basins and their biodiversity. These insights position us to evaluate how biotic events in *Hydrocynus* interfaced with the geological evolution of Neogene Africa.

**Figure 6:**
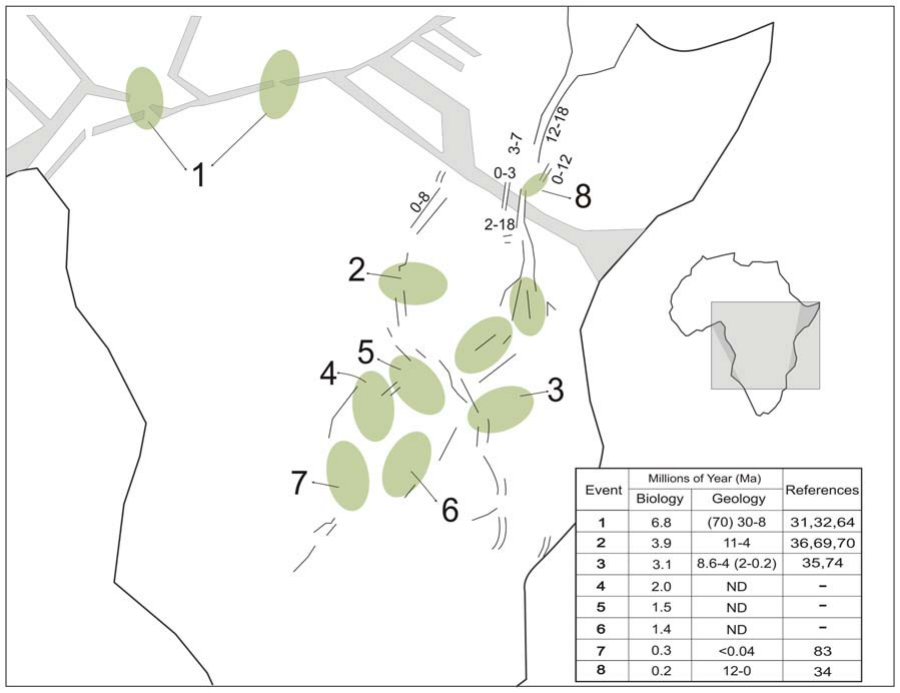
The spatio-temporal congruence of cladogenic events in *Hydrocynus* with episodes of tectonism in the African Rift System. Schematic diagram of south-central Africa illustrating geobiotic regions that delimit the likely locations where antecedent drainage links were tectonically disrupted during rifting and uplift (numbered green ellipses). These events altered boundaries between major drainage basins. Cladogenic events in tigerfishes, constrained by molecular dating of mtDNA sequences, provide biochronologic markers as proxy indicators of these geotectonic events for a subset of rifts (black lines). The inset compares our derived biochronology against the most reliable geochronology derived from the geological literature (numbers denote referenced sources) pertinent to these geobiotic regions. Note that in the Late Cretaceous (66–120 Ma), the Central African Rift System stretched across central Africa from the Atlantic to the Indian Oceans during the initial opening phases of these two oceans (pale gray), but today is only active in west-central Africa, along the Cameroon Line [105], [106]. In East Africa, this early rift phase (Anza Rift) terminated before the onset of the transecting and active East African Rift System (EARS). Here refined geological dates constrain tectonism between 4–12 Ma (focused at ca. 5.5 Ma; see table of cited authorities on Figure), propagating southwards from the Afar Plateau [107]. The new biochronology, derived from *Hydrocynus* evolution, also supports a young and active incipient phase of southwest rift-propagation across the Kalahari Plateau, as has been inferred by geologists for more than 70 years but without firm geochronological evidence. A lacustrine radiation of *Synodontis* catfishes constitutes biochronological evidence for Early Pleistocene initiation of tectonism 1.4>0.9<0.4 Ma [12], [71] in the Okavango graben (Event 7). See text for additional discussion and references.

A mid-Miocene divergence event at 11.1 (CI: 15.5–7.1) Ma separates *H. goliath* from all other *Hydrocynus* species. The cladogenic event that founded *H. brevis* and the Forskahlii complex at 6.8 (CI: 10.8–3.2) Ma, in the late Miocene and Pliocene) corresponds to isolation of the Niger and Sanaga basins along the northern Congo basin [64], when the youngest geologically-dated tectonic events modified the Cameroon volcano-tectonic rift. This is the last remaining active part of a longer lived Central African Rift System that started in the Cretaceous during the opening phases of the Indian and Atlantic Oceans [31], [32], [65] (Figure 6). Further sampling of *Hydrocynus* is required to reconstruct its evolutionary history in the Nilo-Sahelian basins in adequate detail, and further test the Neogene paleogeographical model [66], [67]. Nevertheless, the cladogenesis that founded Nilo-Sahelian *Hydrocynus* overlaps with molecular dating of a late Miocene divergence in African *Crocodylus*, dated at 8.13 (CI: 5.24–12.64) Ma [68]. Corroborating the *Hydrocynus* fossils in the Chad basin (late Miocene ∼7 Ma) [27], [67], these phylogeographic records constitute independent evidence for a late Miocene occurrence of tigerfishes north of the Congo basin. Molecular dating constrains when the latter were isolated from Congo *Hydrocynus* by drainage rearrangement across the Central African Rift Zone, associated with the Cameroon volcano-tectonic rift (Figures 4 and 6).

The Pliocene cladogenic event at 3.9 (CI: 6.9–1.6) Ma isolated Group A in the Congo basin from the clade today represented by *H. tanzaniae* and the Vittatus complex. Its timing concurs with constraints on activity in the western branch of the active East African Rift System (Figure 6), presently geologically-dated to have started between 4–12 Ma, with an apparent focus around 5.5 Ma [36], [65], [69], [70]. This event in *Hydrocynus* is interpreted as vicariance of a formerly contiguous drainage system that linked the eastern Congo basin to the Tanzanian plateau. Association of this event in *Hydrocynus*, with initiation of the rifting that formed Lake Tanganyika overlaps with estimated timings for three lacustrine radiations: in *Synodontis* catfish in the late Miocene at 5.5 (CI 7.3–4.0) Ma [71], *Mastacembelus* eels at 7–8 (CI 10.6–5.5) Ma [72] and platythelphusid crabs conservatively dated at 3.3–2.5 Ma in the late Pliocene [73]. The disruption of the former east-west drainage system, archived as a cladogenic event in these extant *Hydrocynus*, therefore slightly postdates the geologically-dated tectonic uplift in this region of the East African Rift. Nevertheless, despite significant uncertainty in geological dates, together with the wide error on molecular dates, there is a remarkable agreement between the geological- and biological-clocks to support strong linkages between the tectonic and cladogenic events. Initiation of this rifting that formed Lake Tanganyika has recently been reappraised to ∼5.5 Ma [36], [69], [70], and it constitutes the plausible mechanism that disrupted a former east-west drainage system to isolate the Tanzanian plateau from the eastern Congo basin. The Lukuga and Malagarasi rivers are possibly extant vestiges of the former contiguous river (Figure 3).

Divergence between *H. tanzaniae* and the Vittatus complex in the Pliocene at 3.1 (CI: 5.3–1.3) Ma also slightly postdates the initiation of tectonic tilting in the region north of Lake Malawi (Figure 6) and so can be attributed with confidence to such rifting [35], [74]. Furthermore, this phylogeographic signature in *Hydrocynus* is congruent with cladogenic events in bathyergid molerats. These are represented by two genera: *Fukomys* dated at 3.5 (CI: 3.8–3.2) Ma, and represented in species complexes of the Tanzanian and Kalahari plateau, isolated across the Rukwa-Mbeya region [75]; and a cladogenic event across the Malawi rift in silvery mole-rats (genus *Heliophobius*) is also constrained to the late Pliocene [76]. These phylogeographic records of terrestrial mammals are further congruent with speciation of the spiny eels *Mastocembalus frenatus* and *M. shiranus* in Lakes Tanganyika and Malawi, respectively, at 3.9 (CI: 5.6–2.9) Ma [72]. All subsequent divergence events within the Vittatus complex (2.0–0.5 Ma, Plio-Pleistocene), correspond to a time period of several drainage rearrangements in the Zambian Congo and the Upper Zambezi drainage systems [3], [12], [33], [77], which we infer isolated descendents of an ancestral population of *Hydrocynus*, formerly widespread in the Paleo-Chambeshi drainage system in the Pliocene. These events are interpreted to represent the incipient, but complex propagation of the south west extension of the East African Rift System from eastern Africa across the Kalahari plateau [12], [77]–[83], but cannot be dated geologically with any degree of certainty because reliable geochronological techniques lack suitable volcanic minerals in these sediments. In these circumstances, biological clocks provide a high fidelity record of the tectonic processes related to the propagation of the rift system into the Kalahari Plateau. They offer interesting potential to date more recent episodes of neotectonics across this landscape [12].

Three of these key events are represented in the divergence between the two sister groups (Groups C and B from *H. vittatus* and Group D) estimated at 2.0 (CI: 3.5–0.8) Ma. Groups B and C diverged in the Pleistocene at 1.5 (CI: 2.6–0.5) Ma while *H. vittatus* and Group D split approximately 1.4 (CI: 2.4–0.5) Ma. All these events can be attributed to rearrangements of Upper Zambezi and Paleo-Chambeshi headwaters across northeast Zambia, driven by Plio-Pleistocene tectonism, which ramified from the southern arm of the Albertine Rift across the Bangweulu and Mweru depressions, to extend southwest into the Bulozi (Upper Zambezi) and Okavango graben [12], [84, Goodier et al. unpublished].

Three dated nodes, recovered with strong nodal support, all represent relatively recent Pleistocene divergence events that created significant phylogeographic structure within the Vittatus complex. The first, main divergence event, dated to 0.3 (CI: 0.6–0.1) Ma, separated a lineage comprising all but one of the Congo, Lufubu and Kwango haplotypes from the Lower Zambezi-Shire, the southeast African coastal populations and the remaining Congo-Kwango haplotype. The three other well-supported divergences constrain the origins of four Congo and Kwango haplotypes to approximately 0.2 Ma, all the Okavango and Upper Zambezi haplotypes to 0.1 (CI: 0.3–0.0) Ma, and all but one of the Middle Zambezi, Shire and coastal population haplotypes to 0.1 (CI: 0.2–0.0) Ma. The latter two groups are consistent with those well-supported groups recovered in the two undated Bayesian trees discussed above. Moreover, hierarchical AMOVA and GSI tests of *H. vittatus* underscore the significance of lineage structuring within and across the Congo and Zambezi drainage basins, which testify to more recent Pleistocene events within the Zambezi Basin. These events concur with a model of drainage evolution, centered on the paleo-lake complex in northeast Botswana [25], [77], [85], and confirm that the Victoria Falls has exercised significant control over tigerfish evolution. The finer scaled details represented in these events, including the recent speciation event (0.3 Ma) that founded *H. vittatus s.s*, are explored in detail elsewhere [84, Goodier et al. *unpublished data*].

The geographical isolation of the Lake Chamo population of *H. forskahlii*, in the Main Ethiopian Rift, is tentatively constrained at ∼0.2 Ma (Figure 4, Table S1), which points to late Pleistocene severance of its link from the extant Omo drainage system (Lake Turkana) and the Nile basin (Figures 2 and 6). Lakes Chamo and Turkana were formerly linked across the Chew Bahir graben by the Segen drainage [86], [87], and even more recently, Lake Turkana overtopped into the southern Nile basin (Sudd) during the former's Holocene high stand 0.09 Ma (87,88). There is no precise geochronological evidence constraining when outflow along the Segen link (outlet) from Lake Chamo was impounded (F. H. Brown pers. comm.) to isolate the Turkana basin from the Ririba-Chamo-Abaya basins (Main Ethiopian Rift) (Figure 6), so this cladogenic event represented in *H. forskahlii* constrains a recent closure for this linkage, which is amenable to independent geological testing, as recently applied to reconstruct the paleogeography of the Paleo-Omo river [88].

### Conclusions

Analyses of phylogeographic structuring reveal that tectonism exhibits strident signatures of control over the evolution of *Hydrocynus* across and within Africa's principal drainage basins. Historical evidence archived in mitochondrial *cyt b* sequence data resolves formative events in the evolution of both *Hydrocynus* and African river systems since the mid-Miocene. As represented by extant species, this study points to an origin of the genus in the Congo basin, after which ancestral populations seeded tropical drainage basins across low and high Africa. Phylogeographic signatures of drainage evolution reveal that tigerfishes perform very well as biotic indicators of geomorphic history. High sensitivity of these stenotopic species to even subtle alterations of landscapes by incipient rifting (as demonstrated here across the Kalahari Plateau) is invoked to explain the resolution into paleo-environmental evolution conferred by phylogeographic records of *Hydrocynus*. Considered against fragmented knowledge and the general uncertainty about timings of the geomorphological events that altered Africa's drainage [12], [17], the evidence presented here opens complementary windows onto geological events through the mid-Neogene up to the Holocene, where recent events across the Nilo-Sahelian drainage basins aid in the resolution of early Holocene history. These results provide a glimpse of Pan-African tectonic events that have altered drainage topology across central Africa since the early Neogene. A forthcoming paper reports on a finer-scaled reconstruction of the evolution of the *H. vittatus* complex centred on the Zambezi basin and the Zambian Congo tributaries, together with a more detailed reconstruction of drainage evolution across the Kalahari Plateau [Goodier et al unpublished data]. This congruence between geological evidence and the combined phylogeographic signals (both molecular dating constraints and cladogenic structuring), recovered in extant populations within and across drainage basins, across Africa, constitutes encouraging support for this new bio-chronology. It enables geologists to obtain a more detailed geohistory of rift propagation than hitherto has been possible. In turn, it provides a new insight into the rate of tectonic processes across the south-central African Plate [12]. Such encouraging resolution endorses geobiological studies of coevolving landforms and their biota.

## Materials and Methods

### Sample Collection

Fin clips or muscle tissue were collected from 88 *Hydrocynus* individuals sampled from 12 drainage systems in the study area, preserved in 96% ethanol and stored at room temperature or 4°C until DNA extraction (Table S1).

### mtDNA Isolation, Amplification and Sequencing

DNA was extracted from a small piece of tissue (approx. 2 mm^3^) using the chelex extraction protocol [89] with one modification: samples were digested overnight with Collagenase enzyme (50 mg/ul) prior to digestion with Proteinase K (Fermantas). After centrifugation at 13 200 rpm, the resulting supernatant was processed using the Wizard® SV Gel and PCR Clean-Up System (Promega) as per manufacturer's instructions. The final product was eluted in 100 µl of nuclease-free water.

A combination of primers designed in this study and modified universal PCR primers were used to amplify the *cyt* b region (Table S5). Alignment of available *Hydrocynus* sequences, sequences of closely related characiforms from Genbank [90] and partial *cyt* b sequences [18] were used to design primers. The final *cyt* b fragment was amplified as two parts. Two primer combinations (L14724HycF2 or L14990FishF and H15494HycR2, and L15408Hyc with one of three reverse primers (HycGR2, HycR3 or H15919HycR) were used to amplify the respective, partially overlapping amplicons. The large number of reverse primers used was a result of the variability among *Hydrocynus* species at the region initially selected for primer binding, based on the partial Genbank sequences and related characiformes.

For all primer pairs used to amplify the *cyt* b region, the PCR reaction mixture per sample (total volume 50 µl) was composed of 0.5 U SuperTherm *Taq* (Southern Cross Biotech), 200 µM dNTP's, 1× SuperTherm reaction buffer, 2 mM MgCl2, 0.5 µM of each primer, 1 mg/µl BSA and 10 to 50 ng of template DNA. Approximately 20 µl of mineral oil was added to each sample as an overlay. Reactions were done on a Programmable Thermal Controller 100 (MJ Research, Inc.) with the following PCR conditions: 94°C for 5 min; 5 cycles of amplification of 94°C for 40 sec, 48°C for 50 sec and 72°C for 1 min; 30 cycles of amplification of 94°C for 40 sec, 50°C for 50 sec and 72°C for 1 min; and a final extension step of 72°C for 10 min.

PCR products were electrophoresed on 2% agarose gels (1× TBE buffer) with O'Gene Ruler 1 kb DNA Ladder (Fermentas). The target band was excised from the gel and purified using a Wizard® SV Gel and PCR Clean-Up System column (Promega) as per manufacturer's instructions. The purified DNA fragments (10–20 ng of purified PCR product) were sequenced in both directions using the BigDye® Terminator v3.1 Cycle Sequencing Kit (Applied Biosystems). Reactions were performed on an ABI 2700 thermocycler (Applied Biosystems) with the following conditions: 25 cycles of 94°C for 30 sec, 50°C for 5 sec and 60°C for 4 min. To remove unincorporated nucleotides from the final sequence product, 0.5% SDS was added to the sequencing reactions followed by 10 minutes at 95°C and 5 minutes at 25°C. Sequencing reactions were electrophoresed on an ABI 3100 sequencer (Applied Biosystems).

### Data Analyses

DNA sequences were edited using Chromas 2.13 [91] and aligned with the ClustalW algorithm [92] in Bioedit 7.0.5.2 [93], adjusted by eye where necessary. The identity of all sequences was checked using the BLASTn search in the NCBI nucleotide database on Genbank [90], and have been submitted to Genbank (JF703694–JF703783).

Paup 4.0b10 [43] and ModelTest 3.06 [94] were used to establish the best-fit model of nucleotide substitution for the sequence data sets as determined by the hierarchical likelihood ratio tests (hLRT). The General Time Reversible (GTR) model [39] plus Gamma (G) was chosen for the *cyt b* data. Lists of unique sequences (haplotypes) for the *cyt b* data set were generated using Collapse 1.2 [95] (Table S1).

Phylogenies of *Hydrocynus* using the *cyt b* data set were constructed in BEAST 1.4.8 package [41], MrBayes 3.1.1 [42] and PAUP 4.0b10 [43], using the GTR model parameters for each data set. However, only the BEAST data are presented and discussed. BEAST uses Bayesian inference and a MCMC sampling procedure to reconstruct a phylogeny by estimating the probability distribution given sequence data [41], [42]. This algorithm weights trees in proportion to their posterior probability, such that a branch with a posterior probability close to 1 is considered well supported, whereas a value close to 0 is considered very weakly supported.

In BEAST, all starting trees were constructed using the Unweighted Pair Group Method with Arithmetic Mean (UPGMA) [96]. The GTR model of nucleotide evolution and its parameters were specified and the uncorrelated lognormal relaxed molecular clock was used to allow for independent substitution rates on different branches. Short runs of 500 000 generations were performed to optimise the operators, which allows the algorithm to sample the target distribution faster and more efficiently, using the suggestions of the previous run in the following run until no more suggestions were made. A long run of 50 000 000 generations was then performed, logging parameters every 1 000 generations. Burnin was set to the first 5 000 logged trees. All other priors and operators were kept at the default settings. The methods of dating estimation using BEAST are presented at the end of this section.

*Micralestes acutiden*s (Genbank accession number: AY791418.1) and *Ladigesia roloffi* (AY791417.1) were selected as outgroups due to their close genetic relationship to *Hydrocynus* identified by Calcagnotto et al. [18]. Taxa were accepted as representing a certain species based on their affinities to known sequences from respective species from known localities, where available, and to sequences from topotypical material (obtained from the type locality), and identified voucher specimens. For example, *H. vittatus* from the Okavango Delta is considered topotypical [55], [56].

In order to estimate the ages of the lineage divergences of *Hydrocynus*, a relaxed phylogenetics method was implemented in BEAST 1.4.8 [41]. This method employs a relaxed molecular clock that co-estimates the tree and dates of divergence of the lineages, given the stipulated model of nucleotide substation (GTR). An uncorrelated lognormal relaxed molecular clock prior with a Species Birth-death tree prior was used to estimate the timing of divergences between lineages [97]. Short runs of 100 000 generations were performed to optimise the operators, which allows the algorithm to sample the target distribution faster and more efficiently, using the suggestions of the previous run in the following run until no more suggestions were made. A long run of 50 000 000 generations was then performed, logging parameters every 1 000 generations. All other priors and operators were kept at the default settings. Burnin was set to the first 5 000 logged trees. The minimum age of appearance of *Hydrocynus* at approximately 12 Ma [22], [26] was used as a calibration point of the root of the tree with a lognormal distribution. As there is no *Hydrocynus* specific *cyt b* evolutionary rate, a generalised teleost *cyt b* substitution rate of 0.76–2.2% per million years was used as a uniform prior [98]. All other priors and operators were kept at the default settings.

### Nucleotide and Haplotype Diversity and Genetic Divergence between Lineages

Genetic diversity was characterized by the haplotype and nucleotide diversities estimated in Arlequin 3.11 [46]. Rate heterogeneity among sites was corrected for with the gamma shape parameter.

### Population Structure and Demographic History

A measure of genealogical sorting is important to assess differentiation of genetic variation between and within species. The genealogical sorting index (GSI) [47] quantifies the degree of exclusive ancestry of groups of individuals on a rooted phylogenetic tree. This statistic was used to quantify the common ancestry of *cyt b* sequences of *Hydrocynus* in an interspecific context. A GSI value of 1 represents a monophyletic group, while a group of completely mixed genealogical ancestry will have a GSI value of 0. Beyond qualitative assessments of lineage history and orthodox statistical tests of nodal support (including bootstrapping) in phylogenetic reconstruction [47], GSI is an informative statistic because it enables explicit testing of roles of hypothesized paleo-environmental controls (in this study drainage rearrangements and tectonism), by determining the levels of differentiation among lineages; and since individuals can be assigned to groups on criteria of sampling locality, GSI enables structured tests of candidate determinants of the history of different populations. The null hypothesis will expect genealogical admixture across lineages that manifest in low GSI statistics, so correctly rejecting inappropriate candidates as biogeographical determinants. All analyses were run on the GSI website [99]. The groups in the assignment file for the *cyt b* GSI analysis were set according to species and evolutionary lineage and, in order to test for structuring by drainage system, *H. vittatus* samples were grouped based on the drainage system within which they were sampled. A Newick version of the BEAST tree was used in the analysis. The number of bootstrap replications conducted was 10 000.

To test whether extant drainage patterns have influenced extant population structure, an analysis of molecular variance (AMOVA) was calculated in Arlequin 3.11 [48]. This analysis calculates the fixation index (ΦST) [49] which uses haplotype diversities to estimate levels of genetic diversity and differentiation between contrasted populations. ΦST analysis partitions the total genetic variance into among- and within-population components, with population specific ΦST's calculated. To test if genetic variation has been structured within drainage basins, versus across watersheds, and principal knickpoints, a hierarchical Analysis of Molecular Variance (AMOVA) was also performed using Arlequin 3.1 [48] to determine how extant genetic variation is partitioned with *H. vittatus*. This analysis was only performed on this species due to constraints of sample number. Population groupings were based on the overall drainage system (Congo vs. Zambezi and Coastal - A), the drainage system subdivided by separation (Congo vs. Zambezi vs. Coastal - B), and the drainage subdivided by any drainage barriers (Congo vs. Upper Zambezi and Okavango vs. Middle and Lower Zambezi vs. Coastal - C).

Tajima's D [50] and Fu's Fs [51] statistics test for deviations from the neutral theory model for a population of constant size [52]. Tajima's D examines whether the number of polymorphic sites correlates with the average number of nucleotide differences in the data set. Fu's Fs considers, given the number of observed haplotypes, whether it is probable that a larger number exists [53]. In the event of population growth or background selection, larger numbers of low-frequency alleles would be expected to be encountered if the population size was constant, and this would result in a large negative value for the neutrality test. Fu's Fs is particularly sensitive to the signals of recent population expansions [51].

Mismatch distributions [100], [101], [102], the distributions of the observed number of differences between pairs of haplotypes, were used to quantify historical changes in population size in the *Hydrocynus* lineages. A unimodal (‘smooth’) distribution indicates recent population growth compared to a multimodal (‘ragged’) distribution which indicates demographic stability. Here Harpending's ‘raggedness’ index (r) [101] quantifies the “smoothness” of the distribution. Mismatch distributions were only calculated for lineages that showed a significantly negative Tajima's D [50] and/or Fu's Fs [51]. Tajima's D, Fu's Fs and the mismatch analyses were only performed on sample groups with four or more individuals. The AMOVA, Tajima's D, Fu's Fs and the mismatch analyses were carried out in Arlequin 3.11 [46] with 10 000 bootstrap replications conducted for each analysis.

## Supporting Information

Figure S1 **Bayesian tree of the cytochrome b sequence data of** ***Hydrocynus*** **produced in BEAST, using the GTR parameters specified by Modeltest.**
(DOC)

Figure S2 **Bayesian tree of the cytochrome b sequence data of** ***Hydrocynus*** **produced in MrBayes, using the GTR parameters specified by Modeltest.**
(DOC)

Figure S3 **Maximum parsimony of the cytochrome b sequence data of** ***Hydrocynus*** **produced in PAUP, using the GTR parameters specified by Modeltest.**
(DOC)

Table S1 **Summary of 88 genotyped individuals of** ***Hydrocynus*** **characterized in this study, together with 2 Genbank sequences, ordered by taxa with corresponding haplotype designations (total = 42) and their collection sites.**
(DOC)

Table S2 **Descriptive statistics for the** ***cyt b*** **data set that includes all 88 genotypes characterized in this study and 2 additional sequences from Genbank for a total of 42 haplotypes.**
(DOCX)

Table S3 **Table of lineage specific ΦST values.**
(DOC)

Table S4 **Table of lineage specific Tajima's D and Fu's** ***Fs*** **values.**
(DOC)

Table S5 **Sequences (5′ to 3′) of** ***Cytochrome b*** **primers.**
(DOC)
