# Supplementary material for: Cryptic Diversity of African Tigerfish (Genus Hydrocynus) Reveals Palaeogeographic Signatures of Linked Neogene Geotectonic Events
Source: PLoS One. 2011 Dec 14;6(12):e28775. doi: 10.1371/journal.pone.0028775 (PMC3237550; doi:10.1371/journal.pone.0028775)
Supplement: Figure S1 — Bayesian tree of the cytochrome b sequence data of Hydrocynus produced in BEAST, using the GTR parameters specified by Modeltest. (DOC) [file pone.0028775.s001.doc]

**Figure S1 – Bayesian tree of the cytochrome b sequence data of *Hydrocynus* produced in BEAST, using the GTR parameters specified by Modeltest**

**
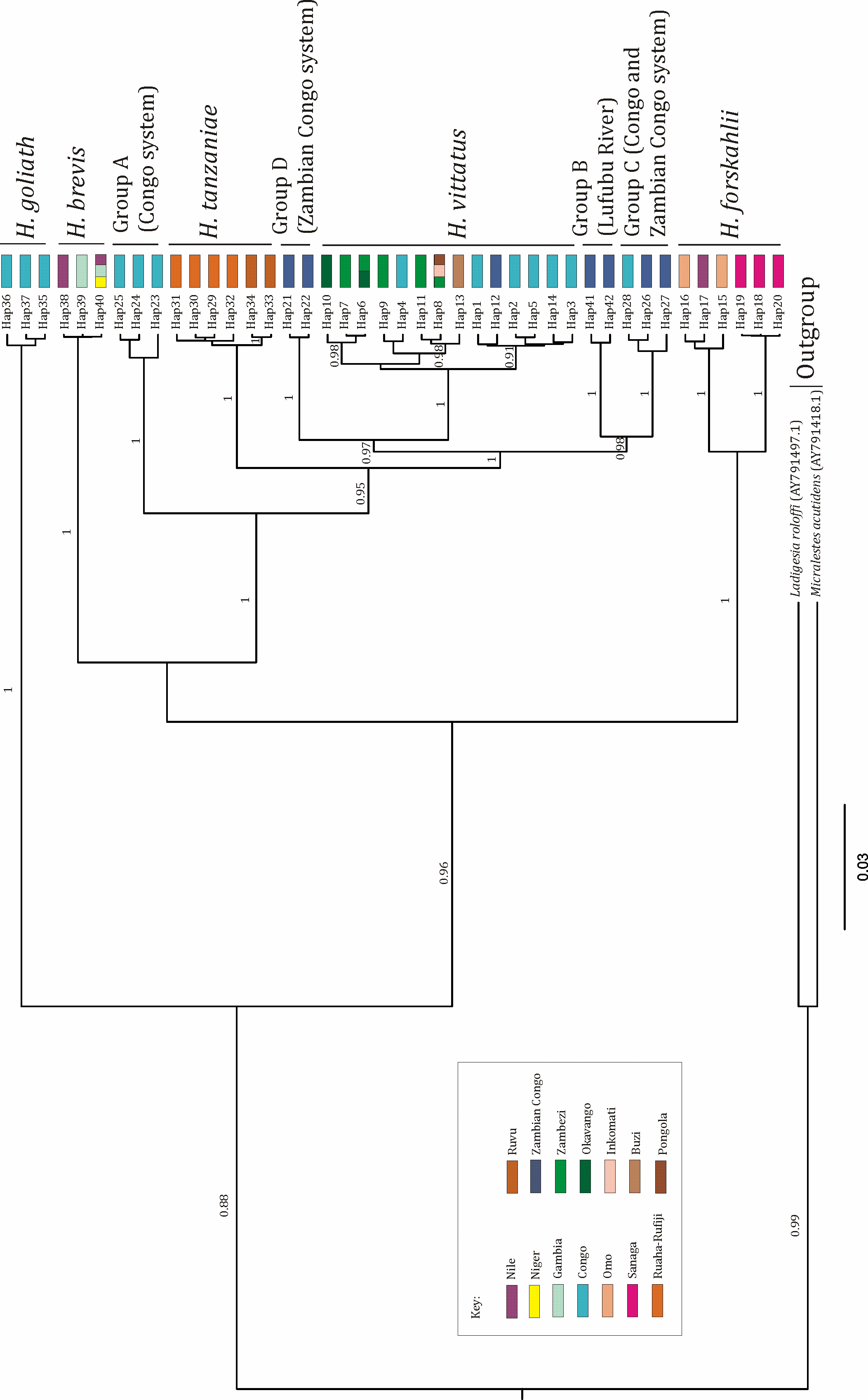
**
