# Supplementary material for: Cryptic Diversity of African Tigerfish (Genus Hydrocynus) Reveals Palaeogeographic Signatures of Linked Neogene Geotectonic Events
Source: PLoS One. 2011 Dec 14;6(12):e28775. doi: 10.1371/journal.pone.0028775 (PMC3237550; doi:10.1371/journal.pone.0028775)
Supplement: Table S1 — Summary of 88 genotyped individuals of Hydrocynus characterized in this study, together with 2 Genbank sequences, ordered by taxa with corresponding haplotype designations (total = 42) and their collection sites. (DOC) [file pone.0028775.s004.doc]

**Table S1 – Summary of 88 genotyped individuals of *Hydrocynus* characterized in this study, together with 2 Genbank sequences, ordered by taxa with corresponding haplotype designations (total = 42) and their collection sites**

| **Sample** | ***Cyt b* Haplotype No.** | **Species** | **Country** | **River/Lake** |
| --- | --- | --- | --- | --- |
|  |  |  |  |  |
| Brev 1 | Hap 38 | *H. brevis* | Sudan | Nile River |
| Brev 2 | Hap 40 | *H. brevis* | Sudan | Nile River |
| Brev 3 | Hap 40 | *H. brevis* | Sudan | Nile River |
| Brev 5 | Hap 40 | *H. brevis* | Sudan | Nile River |
| Brev 7 | Hap 38 | *H. brevis* | Sudan | Nile River |
| Gam 1 | Hap 39 | *H. brevis* | Senegal | Gambia River |
| Gam 2 | Hap 40 | *H. brevis* | Senegal | Gambia River |
| Gam 3 | Hap 40 | *H. brevis* | Senegal | Gambia River |
| AY791400 | Hap 40 | *H. brevis* | Mali | Niger River |
| Ethio 1 | Hap 15 | *H. forskahlii* | Ethiopia | Lake Chamo |
| Ethio 2 | Hap 16 | *H. forskahlii* | Ethiopia | Lake Chamo |
| Ethio 3 | Hap 16 | *H. forskahlii* | Ethiopia | Lake Chamo |
| SUD 3 | Hap 17 | *H. forskahlii* | Sudan | White Nile River |
| CAR 1 | Hap 35 | *H. goliath* | Central African Republic | Congo River |
| Co 1 | Hap 35 | *H. goliath* | Congo | Congo River |
| Co 6 | Hap 36 | *H. goliath* | Congo | Congo River |
| Co 207 | Hap 35 | *H. goliath* | Congo | Congo River |
| Hv 200 | Hap 35 | *H. goliath* | Congo | Congo River |
| Hv 205 | Hap 35 | *H. goliath* | Congo | Congo River |
| INGA 1 | Hap 37 | *H. goliath* | Congo | Congo River |
| Kw 2a | Hap 36 | *H. goliath* | Congo | Kwango River |
| Ruv 1 | Hap 33 | *H. tanzaniae* | Tanzania | Ruvu River |
| Ruv 2 | Hap 33 | *H. tanzaniae* | Tanzania | Ruvu River |
| Ruv 3 | Hap 34 | *H. tanzaniae* | Tanzania | Ruvu River |
| Tan 1 | Hap 29 | *H. tanzaniae* | Tanzania | Rufiji River |
| Tan 2 | Hap 30 | *H. tanzaniae* | Tanzania | Rufiji River |
| Tan 3 | Hap 30 | *H. tanzaniae* | Tanzania | Rufiji River |
| Tan 5 | Hap 31 | *H. tanzaniae* | Tanzania | Rufiji River |
| Tan 10 | Hap 30 | *H. tanzaniae* | Tanzania | Rufiji River |
| Tan 13 | Hap 30 | *H. tanzaniae* | Tanzania | Rufiji River |
| Tan 17 | Hap 32 | *H. tanzaniae* | Tanzania | Rufiji River |
| AY791404 | Hap 14 | *H. vittatus* | Central African Republic | Congo River |
| Bu 1 | Hap 13 | *H. vittatus* | Mozambique | Buzi River |
| CK 2 | Hap 8 | *H. vittatus* | South Africa | Inkomati River |
| CK 8 | Hap 8 | *H. vittatus* | South Africa | Inkomati River |
| Co 2 | Hap 4 | *H. vittatus* | Congo | Congo River |
| Co 3 | Hap 3 | *H. vittatus* | Congo | Congo River |
| DRS 4 | Hap 6 | *H. vittatus* | Botswana | Okavango River |
| Gum 1 | Hap 6 | *H. vittatus* | Botswana | Okavango River |
| Gum 4 | Hap 10 | *H. vittatus* | Botswana | Okavango River |
| Hv 202 | Hap 2 | *H. vittatus* | Congo | Congo River |
| Hv 203 | Hap 1 | *H. vittatus* | Congo | Congo River |
| Kw 3a | Hap 5 | *H. vittatus* | Congo | Kwango River |
| Kw 50 | Hap 4 | *H. vittatus* | Congo | Kwango River |
| Kw 214 | Hap 4 | *H. vittatus* | Congo | Kwango River |
| Mz 12 | Hap 11 | *H. vittatus* | Zimbabwe | Lake Kariba |
| Mz 14 | Hap 8 | *H. vittatus* | Zimbabwe | Lake Kariba |
| Nxa 9 | Hap 6 | *H. vittatus* | Botswana | Okavango River |
| Sh 2 | Hap 9 | *H. vittatus* | Malawi | Lower Shire River |
| Sh 7 | Hap 8 | *H. vittatus* | Malawi | Lower Shire River |
| Tang 1 | Hap 12 | *H. vittatus* | Zambia | Lake Tanganyika |
| TanLu 1 | Hap 12 | *H. vittatus* | Zambia | Lufubu River |
| TanLu 3 | Hap 12 | *H. vittatus* | Zambia | Lufubu River |
| TanLu 4 | Hap 12 | *H. vittatus* | Zambia | Lufubu River |
| TanLu 6 | Hap 12 | *H. vittatus* | Zambia | Lufubu River |
| TanLu 10 | Hap 12 | *H. vittatus* | Zambia | Lufubu River |
| Us 2 | Hap 8 | *H. vittatus* | Swaziland | Usuthu River |
| Us 3 | Hap 8 | *H. vittatus* | Swaziland | Usuthu River |
| UZ 50 | Hap 6 | *H. vittatus* | Zambia | Upper Zambezi River |
| UZ 56 | Hap 6 | *H. vittatus* | Zambia | Upper Zambezi River |
| UZ 61 | Hap 6 | *H. vittatus* | Zambia | Upper Zambezi River |
| UZ 70 | Hap 6 | *H. vittatus* | Zambia | Upper Zambezi River |
| UZ 71 | Hap 7 | *H. vittatus* | Zambia | Upper Zambezi River |
| Co 7 | Hap 25 | Group A | Congo | Congo River |
| Co 171 | Hap 24 | Group A | Congo | Congo River |
| Co 172 | Hap 24 | Group A | Congo | Congo River |
| Kw 4a | Hap 23 | Group A | Congo | Kwango |
| Tshib 1 | Hap 24 | Group A | Congo | Lulua River |
| TanLu 5 | Hap 42 | Group B | Zambia | Lufubu River |
| TanLu 21 | Hap 41 | Group B | Zambia | Lufubu River |
| Bant 1 | Hap 26 | Group C | Zambia | Lake Bangweulu |
| Bant 2 | Hap 26 | Group C | Zambia | Lake Bangweulu |
| Cam 1 | Hap 28 | Group C | Cameroon | Dja River |
| Mw 5 | Hap 27 | Group C | Zambia | Luapula River |
| Mw 16 | Hap 26 | Group C | Zambia | Luapula River |
| Mw 29 | Hap 26 | Group C | Zambia | Luapula River |
| Ban 6 | Hap 21 | Group D | Zambia | Lake Bangweulu |
| Ban 13 | Hap 21 | Group D | Zambia | Lake Bangweulu |
| BanS 1 | Hap 21 | Group D | Zambia | Lake Bangweulu |
| BanS 3 | Hap 21 | Group D | Zambia | Lake Bangweulu |
| Cham 5 | Hap 22 | Group D | Zambia | Chambeshi River |
| Kasenga 1 | Hap 21 | Group D | Zambia | Luapula River |
| Kasenga 2 | Hap 21 | Group D | Zambia | Luapula River |
| Mw 6 | Hap 26 | Group D | Zambia | Luapula River |
| Mw 19 | Hap 21 | Group D | Zambia | Luapula River |
| Mw 25a | Hap 21 | Group D | Zambia | Luapula River |
| Saca 1 | Hap 19 | Group E | Cameroon | Sanaga River |
| Saca 2 | Hap 20 | Group E | Cameroon | Sanaga River |
| Saca 3 | Hap 19 | Group E | Cameroon | Sanaga River |
| Saca 5 | Hap 18 | Group E | Cameroon | Sanaga River |
