# Supplementary material for: Cryptic Diversity of African Tigerfish (Genus Hydrocynus) Reveals Palaeogeographic Signatures of Linked Neogene Geotectonic Events
Source: PLoS One. 2011 Dec 14;6(12):e28775. doi: 10.1371/journal.pone.0028775 (PMC3237550; doi:10.1371/journal.pone.0028775)
Supplement: Table S2 — Descriptive statistics for the cyt b data set that includes all 88 genotypes characterized in this study and 2 additional sequences from Genbank for a total of 42 haplotypes. (DOCX) [file pone.0028775.s005.docx]

## Table S2 – Table of the descriptive statistics for the *cyt b* data set that includes all 88 genotypes characterized in this study and 2 additional sequences from Genbank for a total of 42 haplotypes

|  | **Number of**  **individuals** | **Number of haplotypes** | **Haplotype**  **diversity**  **(Hd)** | **Nucleotide**  **diversity** |
| --- | --- | --- | --- | --- |
| ***H. vittatus*** | 32 | 14 | 0.8911 ±0.0312 | 0.0031 ±0.0019 |
| ***H. goliath*** | 8 | 3 | 0.6071 ±0.1640 | 0.0008 ±0.0008 |
| ***H. forskahlii*** | 4 | 3 | 0.8333 ±0.2224 | 0.0035 ±0.0027 |
| ***H. brevis*** | 9 | 3 | 0.5556 ±0.1653 | 0.0008 ±0.0007 |
| **A** | 5 | 3 | 0.7000 ±0.2184 | 0.0020 ±0.0016 |
| **B** | 2 | 2 | 1.0000 ±0.5000 | 0.0037 ±0.0043 |
| **C** | 7 | 3 | 0.5238 ±0.2086 | 0.0007 ±0.0007 |
| **D** | 9 | 2 | 0.2222 ±0.1662 | 0.0000 ±0.0000 |
| **E** | 4 | 3 | 0.8333 ±0.2224 | 0.0012 ±0.0012 |
| ***H. tanzaniae*** | 10 | 6 | 0.8444 ±0.1029 | 0.0046 ±0.0029 |
| **TOTALS** | 90 | 42 |  |  |
