# Supplementary material for: Cryptic Diversity of African Tigerfish (Genus Hydrocynus) Reveals Palaeogeographic Signatures of Linked Neogene Geotectonic Events
Source: PLoS One. 2011 Dec 14;6(12):e28775. doi: 10.1371/journal.pone.0028775 (PMC3237550; doi:10.1371/journal.pone.0028775)
Supplement: Table S3 — Table of lineage specific ΦST values. (DOC) [file pone.0028775.s006.doc]

## Table S3 – Table of lineage specific ΦST values

|  | **ΦST** |
| --- | --- |
| ***H. vittatus*** | 0.9749 |
| ***H. goliath*** | 0.97614 |
| ***H. forskahlii*** | 0.95562 |
| ***H. brevis*** | 0.97614 |
| ***H. tanzaniae*** | 0.97425 |
| **A** | 0.97567 |
| **B** | 0.97552 |
| **C** | 0.97621 |
| **D** | 0.97654 |
