# Supplementary material for: Cryptic Diversity of African Tigerfish (Genus Hydrocynus) Reveals Palaeogeographic Signatures of Linked Neogene Geotectonic Events
Source: PLoS One. 2011 Dec 14;6(12):e28775. doi: 10.1371/journal.pone.0028775 (PMC3237550; doi:10.1371/journal.pone.0028775)
Supplement: Table S4 — Table of lineage specific Tajima's D and Fu's Fs values. (DOC) [file pone.0028775.s007.doc]

## Table S4 Table of lineage specific Tajima’s D and Fu’s *Fs* values

|  | **Tajima's *D*** | **Fu's *Fs*** |
| --- | --- | --- |
| ***H. vittatus*** | -1.264 | -6.2153* |
| ***H. goliath*** | -0.4479 | -0.4776 |
| ***H. forskahlii*** | 1.9978 | 2.9420 |
| ***H. brevis*** | -0.5833 | -0.5321 |
| **A** | -1.0938 | 0.2764 |
| **D** | 0.0000 | -0.2635 |
| **C** | -1.2372 | -0.9218 |
| ***H. tanzaniae*** | 0.0817 | -0.3826 |
| **B** | 0.0000 | 1.0986 |

*: Significant values for p < 0.05
