# Supplementary material for: Cryptic Diversity of African Tigerfish (Genus Hydrocynus) Reveals Palaeogeographic Signatures of Linked Neogene Geotectonic Events
Source: PLoS One. 2011 Dec 14;6(12):e28775. doi: 10.1371/journal.pone.0028775 (PMC3237550; doi:10.1371/journal.pone.0028775)
Supplement: Table S5 — Sequences (5′ to 3′) of Cytochrome b primers. (DOC) [file pone.0028775.s008.doc]

## Table S5 Sequences (5’ to 3’) of *Cytochrome b* primers

| **Primer** | **Sequence (5’ to 3’)** | **Source** |
| --- | --- | --- |
| **H14724Hyc2** | GACCTATGACTTGAAAAACCATCGTTG | Modified from Irwin *et al*. (1991) |
| **L14990FishF** | ATYTCAGCATGATGAAAYTTTGGTM | Modified from Irwin *et al*. (1991) |
| **H15494HycR2** | AARTATCATTCTGGCTTRATATGYGG | Modified from Irwin *et al*. (1991) |
| **L15408HycF** | GAAACAGGCTCCAACAACC | Modified from Irwin *et al*. (1991) |
| **H15915HycR** | TTAACCTTCRATCTTCGGATTACAAGAC | Modified from Irwin *et al*. (1991) |
| **HycGR2** | GTATCTCTTGATGTTATGTCC | Designed for this study |
| **HycR3** | AGAATGCTTTATGTCTACC | Designed for this study |
